# Ultrasound-guided transabdominal microwave ablation versus laparoscopic myomectomy for FIGO type 3–5 uterine fibroids: a retrospective comparison of efficacy and safety

**DOI:** 10.3389/fmed.2026.1905429

**Published:** 2026-08-07

**Authors:** Mengjia Xia, Shuangshuang Zhao, Zheng Zhang, Yanwei Chen, Mengfan Yang, Zheng Hua, Xuechun Huang, Baoding Chen

**Affiliations:** 1 Department of Medical Ultrasound, Affiliated Hospital of Jiangsu University, Zhenjiang, China; 2 Department of Medical Ultrasound, Cancer Institute of Jiangsu University, Affiliated Hospital of Jiangsu University, Zhenjiang, China; 3 Department of Medical Ultrasound, Affiliated Changzhou No. 2 People’s Hospital of Nanjing Medical University, Changzhou, China

**Keywords:** laparoscopic myomectomy, microwave ablation, thermal ablation, ultrasound-guided, uterine fibroid

## Abstract

**Objective:**

This retrospective cohort study aimed to compare the efficacy and safety of ultrasound-guided transabdominal microwave ablation (MWA) and laparoscopic myomectomy (LM) for intramural uterine fibroids (FIGO type 3–5).

**Method:**

Our retrospective study included 60 patients with uterine fibroids undergoing MWA (*n* = 23) or LM (*n* = 37). Patients in these groups were compared for baseline characteristics, symptom improvement, operation time, postoperative hospital stay, medical costs, and postoperative complications. For the MWA group, changes in fibroid volume were also investigated. Intergroup comparisons in continuous variables were performed using the Mann-Whitney U test, and intragroup comparisons were assessed by the Wilcoxon signed-rank test. Categorical variables were compared using the Chi-square or Fisher’s exact test.

**Result:**

At 6-month of follow-up, no statistically significant difference in overall symptom resolution was observed between the MWA and LM groups (*P* = 0.18). Regarding fibroid volume, the most pronounced reduction occurred within the first 6 months, with the median volume decreasing from 96.46 ml at baseline to 44.23 ml at 6 months (*P* < 0.001), representing a median VRR of 58.27%. Thereafter, the reduction rate gradually slowed. When analyzed by FIGO subtype, type 3 fibroids showed the most pronounced reduction at 3 months, whereas type 4 fibroids exhibited the greatest reduction at both the 6- and 12-month evaluations. Although operative time was comparable between groups (*P* = 0.17), MWA was associated with significantly shorter postoperative hospital stay (*P* < 0.001) and lower overall costs (*P* = 0.01). Moreover, no major complications (e.g., adjacent organ injury, massive hemorrhage, or severe infection) occurred in either group. Minor complications occurred in both groups with comparable incidence (*P* = 0.72) and all resolved with symptomatic management.

**Conclusion:**

Both MWA and LM demonstrated comparable short-term efficacy and safety for FIGO type 3–5 fibroids in our cohort. In addition, MWA offered significantly shorter hospital stay and lower treatment costs, suggesting that it may represent a minimally invasive and potentially cost-saving treatment option.

## 1 Introduction

Uterine fibroids represent one of the most prevalent benign tumors among reproductive-aged women (1), with an overall prevalence of 50%–60% (2). Given the high prevalence, the central clinical challenge lies not merely in diagnosis, but in judiciously identifying patients for intervention and formulating personalized management strategies.

Management decisions should integrate clinical symptoms, fibroid characteristics (size, subtype, and location), and fertility intentions. The International Federation of Gynecology and Obstetrics (FIGO) classification helps refine this assessment by linking symptoms to fibroid anatomy. Among various subtypes, intramural fibroids (FIGO type 3–5) are the most clinically significant (3). Type 3 fibroids, contacting the endometrium, typically result in heavy or prolonged menstrual bleeding (4, 5), while type 5 fibroids, situated beneath the serosa, may lead to compressive symptoms like constipation or urinary frequency (5, 6). Such symptomatic burdens constitute a primary indication for treatment.

The management of uterine fibroids primarily involves pharmacological or surgical interventions. Pharmacological treatments, such as hormonal therapy, alleviate uterine fibroid-associated symptoms by inhibiting the stimulatory effects of gonadal steroid hormones on fibroid growth. However, they are often contraindicated in women planning pregnancy and offer only temporary relief (7). Consequently, surgical intervention remains the primary therapeutic approach for patients with clinical symptoms or anxiety regarding fibroid progression (8). Although hysterectomy is a definitive treatment for uterine fibroids (5), the uterus serves functions beyond reproduction, including endocrine regulation, pelvic floor support, and psychosocial significance. This recognition, coupled with advances in minimally invasive techniques, has moved the clinical emphasis toward uterine-preserving strategies. Among these, laparoscopic myomectomy (LM) is the established standard, offering complete fibroid resection (9). However, its inherent invasiveness and associated anesthetic risks, though less than those of open surgery, remain persistent challenges.

In recent years, ultrasound-guided microwave ablation (MWA) has emerged as a promising therapeutic approach. Initially applied to solid tumors in the thyroid, liver, and lung (10–15), its applications have now expanded to include the management of uterine fibroids. This technique achieves *in situ* tumor devitalization via thermal-induced coagulative necrosis (16, 17), thereby reducing fibroid volume and alleviating associated symptoms (18, 19).

Despite the increasing adoption of MWA, direct comparative evidence with LM for intramural fibroids remains limited. Although both techniques preserve the uterus, they differ fundamentally in approach, which may entail different trade-offs in efficacy, safety, recovery, and cost. Moreover, data on long-term outcomes and subtype-specific responses to MWA remain scarce. Accordingly, this study was designed to address these gaps by comparing the two approaches in patients with FIGO type 3–5 fibroids.

## 2 Materials and methods

### 2.1 Patients

**FIGURE 1:**
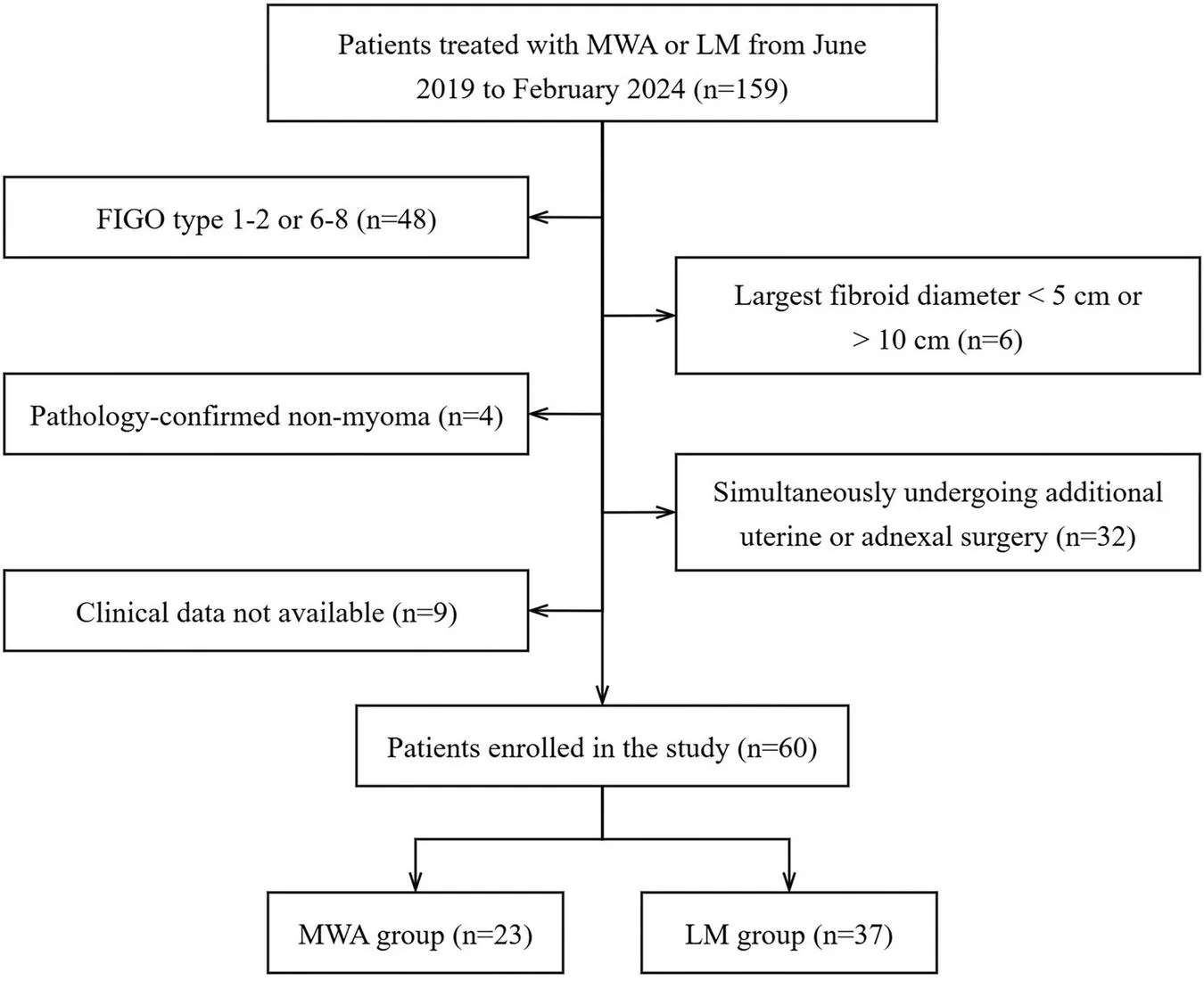
Flowchart of the patient selection process. MWA, microwave ablation; LM, laparoscopic myomectomy; FIGO, International Federation of Gynecology and Obstetrics.

This study conducted a retrospective analysis of medical records from patients who underwent either MWA or LM at the Affiliated Hospital of Jiangsu University and Changzhou NO.2 People’s Hospital from June 2019 to February 2024. Treatment allocation was determined by patient choice following physician consultation. The inclusion criteria were as follows: (1) diagnosis of uterine fibroids by ultrasonography or MRI; (2) the largest fibroid diameter measured 5–10 cm; (3) fibroids classified as FIGO type 3–5. Exclusion criteria comprised: (1) simultaneously undergoing additional uterine or adnexal surgery; (2) pathology-confirmed non-myoma; (3) incomplete clinical data; (4) presence of malignant uterine or adnexal lesions; (5) current pregnancy or lactation; (6) severe systemic or pelvic inflammation; (7) severe dysfunction of major organs; (8) severe coagulation disorders. Ultimately, a cohort of 60 eligible patients was included in the study and categorized into the MWA group (*n* = 23) or the LM group (*n* = 37) based on the therapeutic intervention they underwent. The patient selection flowchart is presented in Figure 1. Our study protocol was approved by the Ethics Committee of the Affiliated Hospital of Jiangsu University (KY2025K0701).

### 2.2 MWA procedure

**FIGURE 2:**
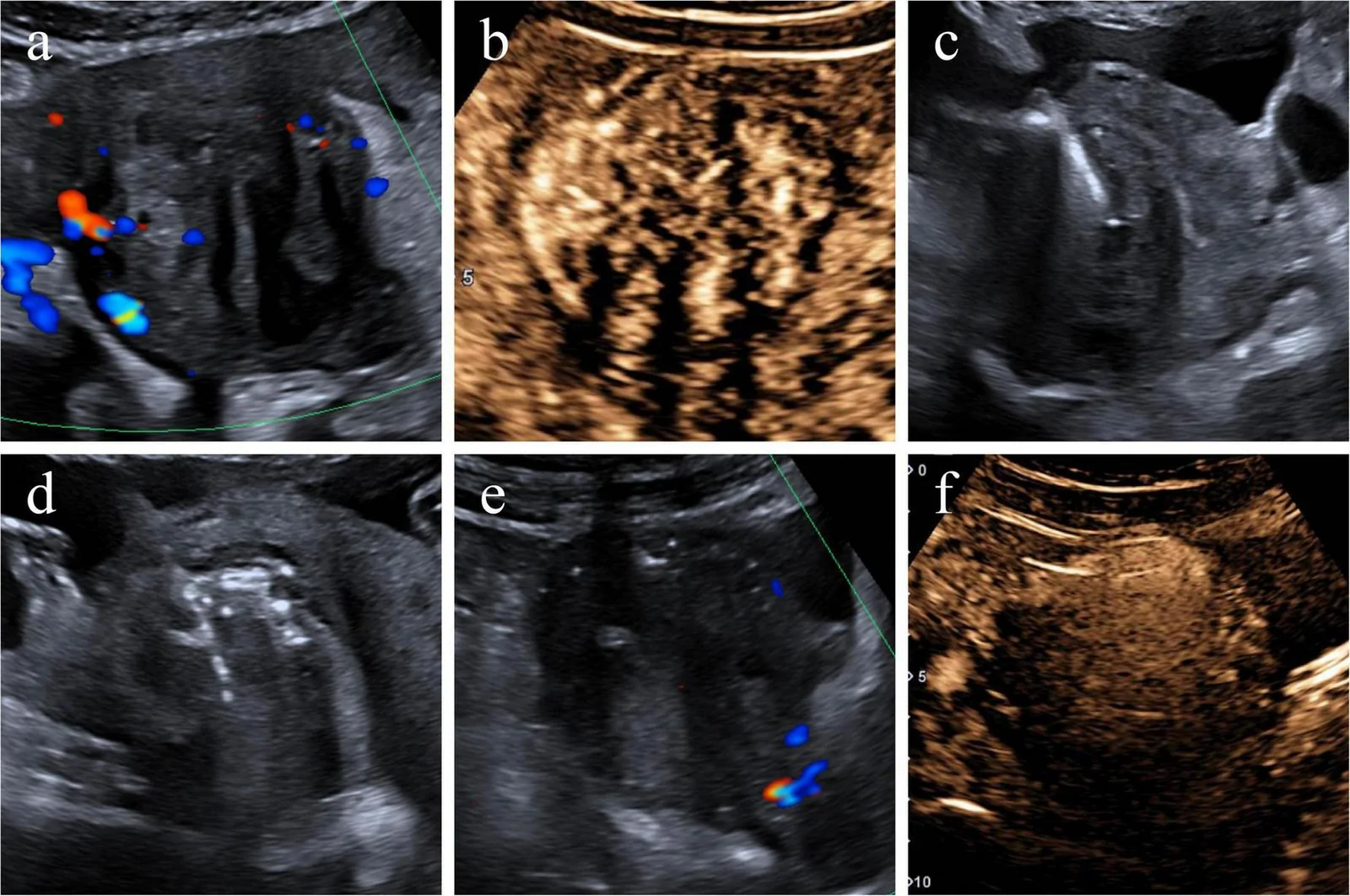
Intraoperative sonographic image during MWA. **(a)** Pre-ablation color Doppler ultrasound demonstrated blood flow signals within and around the fibroid. **(b)** Pre-ablation CEUS revealed pronounced enhancement of the fibroid. **(c)** Pre-ablation needle biopsy. **(d)** Intraoperative ablation zone exhibited hyperechoic changes. **(e)** Post-ablation color Doppler ultrasound showed near-complete disappearance of blood flow signals. **(f)** Post-ablation CEUS exhibited no significant enhancement within the ablation zone.

Patients were placed in the supine position and administered intravenous conscious sedation combined with local anesthesia at the puncture site. Before ablation, artificial ascites was induced by injecting an appropriate amount of 0.9% normal saline into the abdominal cavity to protect surrounding critical organs (such as the bladder, intestines, etc.). Under real-time transabdominal ultrasound guidance, the MWA antenna was percutaneously inserted into the deep portion of the target fibroid, with the tip positioned approximately 0.5 cm from the distal margin. Ablation was performed using the moving-shot technique, whereby the antenna was progressively advanced in a stepwise manner from the posterior to the anterior aspect. Microwave power was set in the range of 45–60 W, with the specific power and ablation duration adjusted based on the individual fibroid characteristics, including size, location, and vascularity. Throughout the procedure, continuous ultrasound monitoring was employed to visualize the antenna position and assess echogenic changes within the fibroid. Contrast-enhanced ultrasound (CEUS) was performed immediately after ablation to evaluate the ablation zone. If residual viable tissue was detected, supplementary ablation was performed during the same session (Figure 2).

### 2.3 LM procedure

Under general anesthesia, the patient was positioned supine. A 1 cm vertical umbilical incision was created, through which a trocar was placed to establish carbon dioxide pneumoperitoneum. A laparoscope was then inserted to examine the abdominal and pelvic cavities. Two ancillary incisions (0.5–1.0 cm each) were made bilaterally in the lower abdomen, medial to the anterior superior iliac spines, to accommodate additional trocars and laparoscopic instruments. Using a unipolar electrode hook, a spindle-shaped incision was made over the fibroid surface, extending into the tumor mass. Subsequently, the fibroid was enucleated in its entirety through a combination of blunt and sharp dissection, and the uterine defect was closed in layers. The resected tissue was morcellated using a power morcellator and extracted via the incision in the left lower abdomen, after which the pelvic cavity was irrigated until the effluent was clear. Upon completion of the surgical procedure, the remaining gas was released, the instruments were withdrawn, and the incisions were sutured.

### 2.4 Follow-up

Symptom improvement was evaluated at the 6-month follow-up based on electronic medical records. For patients in the MWA group, ultrasound examinations (including CEUS when necessary) were conducted at 3, 6, 12, and 24 months postoperatively to monitor changes in fibroid volume. The fibroid volume was calculated using the formula:


V⁢o⁢l⁢u⁢m⁢e=0.52×a×b×c

where a, b, and c represent the three maximum diameters measured in mutually perpendicular planes. The volume reduction rate (VRR) was computed based on serial volumetric assessments as follows:


$$V\,R\,R=\frac{V_{p\,r\,e}-V_{p\,o\,s\,t}}{V_{p\,r\,e}}\times 100\%$$

where V_*pre*_ and V_*post*_ denote the fibroid volumes before and after treatment, respectively. Treatment efficacy was defined as a VRR ≥ 10% at the 3-month follow-up assessment (17).

### 2.5 Statistical analysis

To account for the potential confounding effects of perimenopausal hormonal changes on symptom assessment, age stratification was performed using a cutoff of 46 years, based on The 2023 Chinese Menopause Symptom Management and Menopausal Hormone Therapy Guidelines (20). Statistical analyses were performed with SPSS (version 27.0). Continuous variables were expressed as median and interquartile range (IQR, 25th–75th percentile). Intergroup comparisons were performed using the Mann-Whitney U test, and intragroup comparisons were assessed by the Wilcoxon signed-rank test. Categorical variables, presented as absolute numbers and percentages (n, %), were compared using the Chi-square test or Fisher’s exact test, as appropriate. A *P*-value < 0.05 was defined as statistically significant.

## 3 Results

### 3.1 Comparison of baseline characteristics between the two groups

Baseline characteristics were well balanced between the two groups (Table 1). FIGO type distribution was comparable between groups (*P* = 0.89), with type 4 and type 5 being the most common in both. The median maximum diameter of the treated fibroids was 6.10 cm in the MWA group and 6.00 cm in the LM group (*P* = 0.38), while the median baseline volume was 96.46 ml versus 78.23 ml, respectively (*P* = 0.08).

**TABLE 1:** Baseline characteristics between the two groups.

Characteristics	MWA group	LM group	*P*-value
Patient	23	37	
Age			0.47
<46 years	19 (82.61)	33 (89.19)	
≥46 years	4 (17.39)	4 (10.81)	
Number of treated fibroids^a^	1.00 (1.00, 3.00)	2.00 (1.00, 3.00)	0.40
FIGO classification^b^			0.89
Type 3	4 (17.39)	5 (13.51)	
Type 4	9 (39.13)	14 (37.84)	
Type 5	10 (43.48)	18 (48.65)	
The maximum diameter (cm)^ab^	6.10 (5.50, 7.50)	6.00 (5.50, 6.60)	0.38
Baseline volume (ml)^ab^	96.46 (74.26, 147.55)	78.23 (60.16, 97.81)	0.08
Clinical symptom			
Menorrhagia	6 (26.09)	9 (24.32)	0.88
Prolonged menstruation	3 (13.04)	6 (16.22)	1.00
Irregular menstruation	4 (17.39)	3 (8.11)	0.41
Dysmenorrhea	6 (26.09)	8 (21.62)	0.69
Anemia	6 (26.09)	6 (16.22)	0.51
Pelvic pressure	4 (17.39)	7 (18.92)	1.00

^a^Data are medians with IQRs in parentheses.

^b^Characteristics of the largest treated fibroid. MWA, microwave ablation; LM, laparoscopic myomectomy; FIGO, International Federation of Gynecology and Obstetrics.

### 3.2 Comparison of therapeutic efficacy between the two groups

**FIGURE 3:**
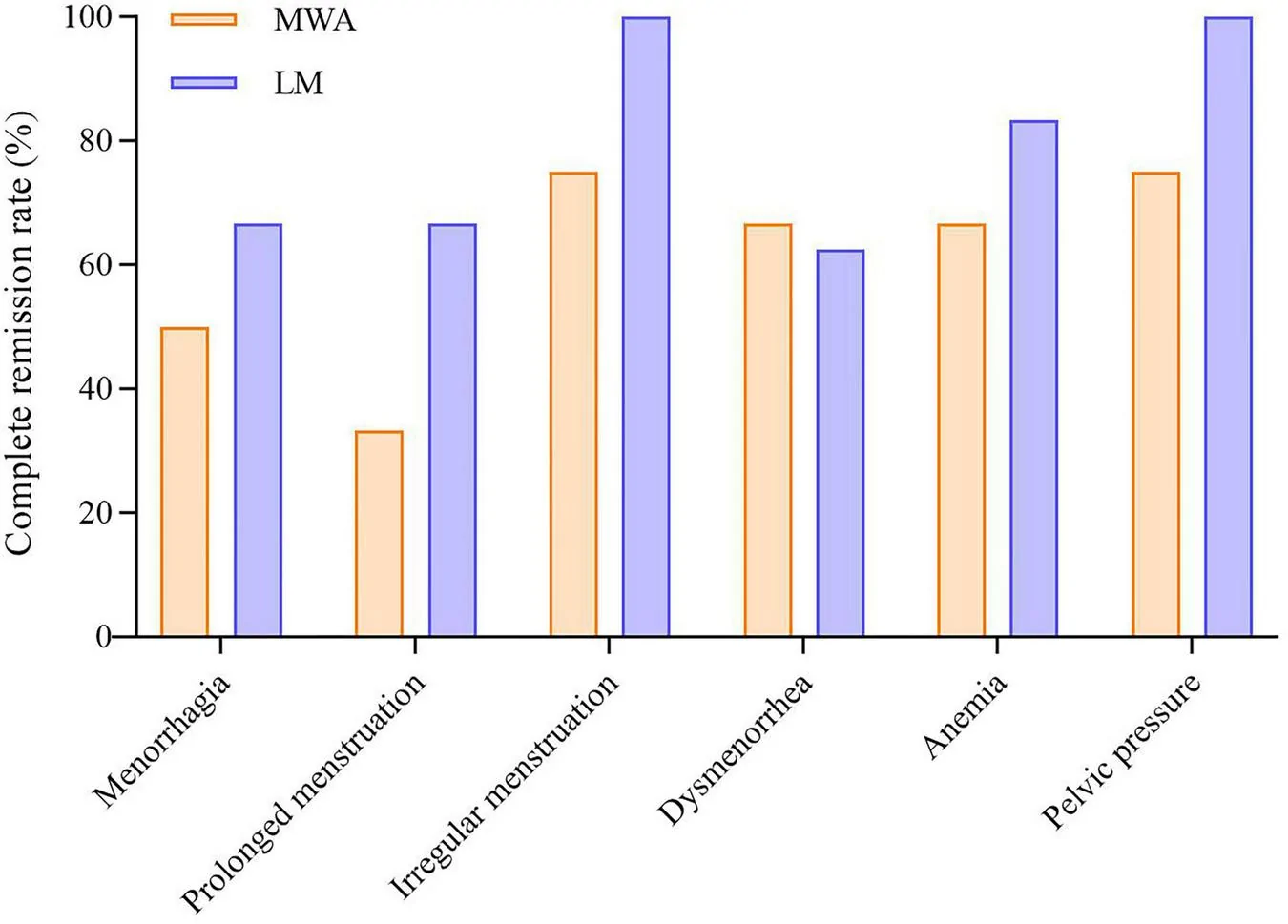
Complete symptom remission rate between the two groups. MWA, microwave ablation; LM, laparoscopic myomectomy.

At the 6-month postoperative follow-up, symptom outcomes ranged from substantial alleviation to complete resolution. Intergroup analysis revealed no statistically significant difference in overall symptom resolution between the MWA and LM groups (18/29 [62.07%] vs. 30/39 [76.92%]; *P* = 0.18). However, trends varied according to symptom type and treatment modality, as detailed in Figure 3.

CEUS performed immediately post-MWA showed no significant enhancement within any treated lesion, indicating an initial technical success rate of 100%. At the 3-month follow-up, based on the criterion of a VRR ≥ 10%, the therapeutic efficacy rate was 95.65% (22/23). During the long-term follow-up, the proportion of patients without ultrasound data at 12 and 24 months postoperatively was 21.74% (5/23) and 73.91% (17/23), respectively (Table 2). Furthermore, two patients underwent additional interventions at 7 months postoperatively.

**TABLE 2:** Volume changes in the MWA group.

Time	Number (*n*)	Volume (ml)^a^	VRR (%)	*P*-value
Baseline	23	96.46 (74.26, 147.55)		
3-month follow-up	23	57.41 (45.86, 84.69)	35.66 (20.27, 53.89)	<0.001
6-month follow-up	23	44.23 (22.90, 59.66)	58.27 (48.50, 73.12)	<0.001
12-month follow-up	18	26.99 (11.98, 52.68)	67.89 (62.20, 88.02)	<0.001
24-month follow-up	6	21.09 (11.58, 48.21)	80.87 (69.55, 89.38)	0.03

^a^Data are medians with IQRs in parentheses. VRR, volume reduction rate.

**FIGURE 4:**
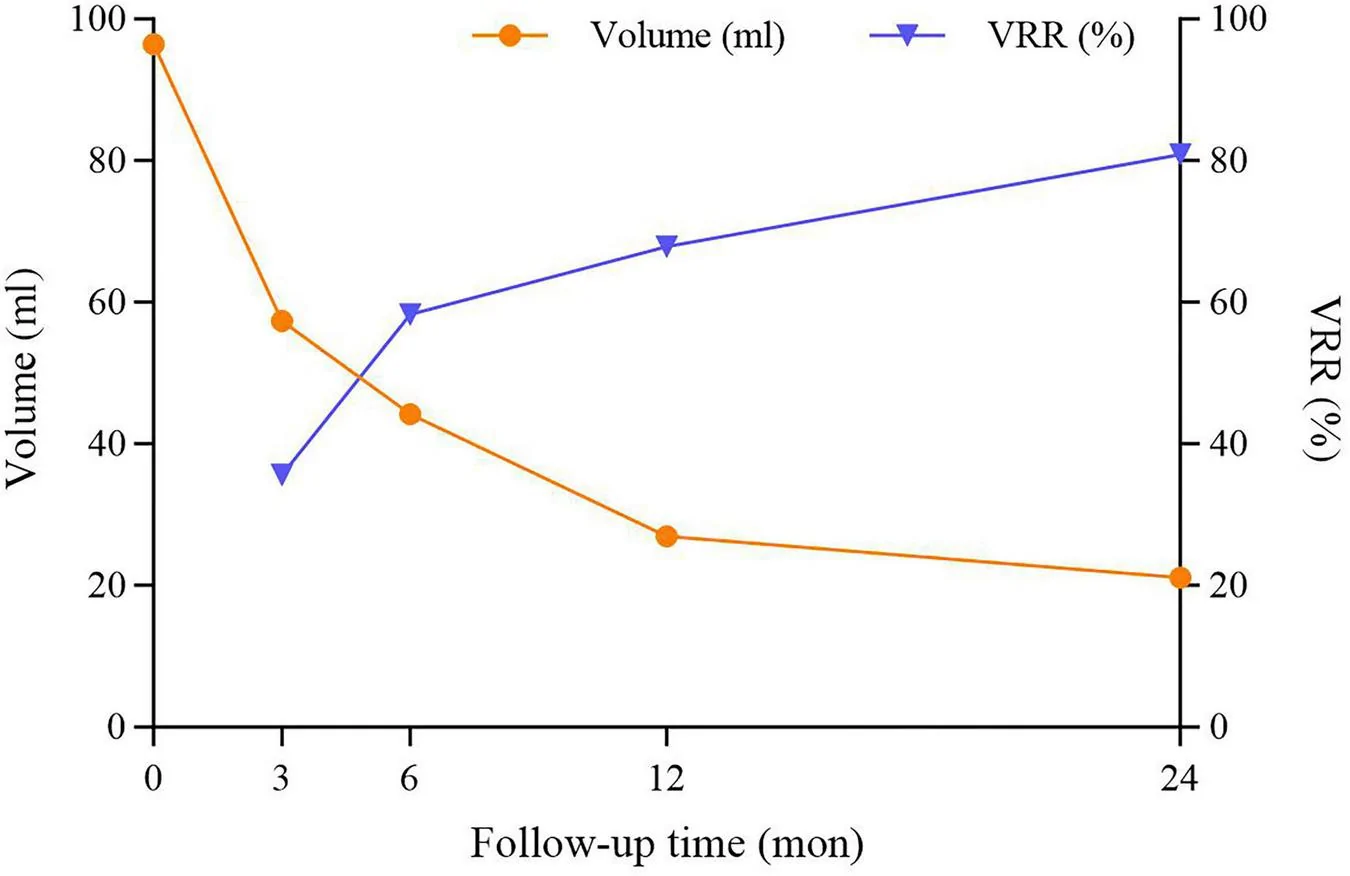
Volume changes in the MWA group. VRR, volume reduction rate.

Following MWA treatment, the volume of uterine fibroids demonstrated a progressive decline over time (Figure 4 and Table 2). The most pronounced reduction occurred during the first 6 months, with the median volume decreasing from 96.46 ml at baseline to 44.23 ml at 6 months (*P* < 0.001), corresponding to a median VRR of 58.27%. The reduction rate gradually slowed thereafter, with the median volume decreasing to 26.99 ml at 12 months (*P* < 0.001) and 21.09 ml at 24 months (*P* = 0.03).

**FIGURE 5:**
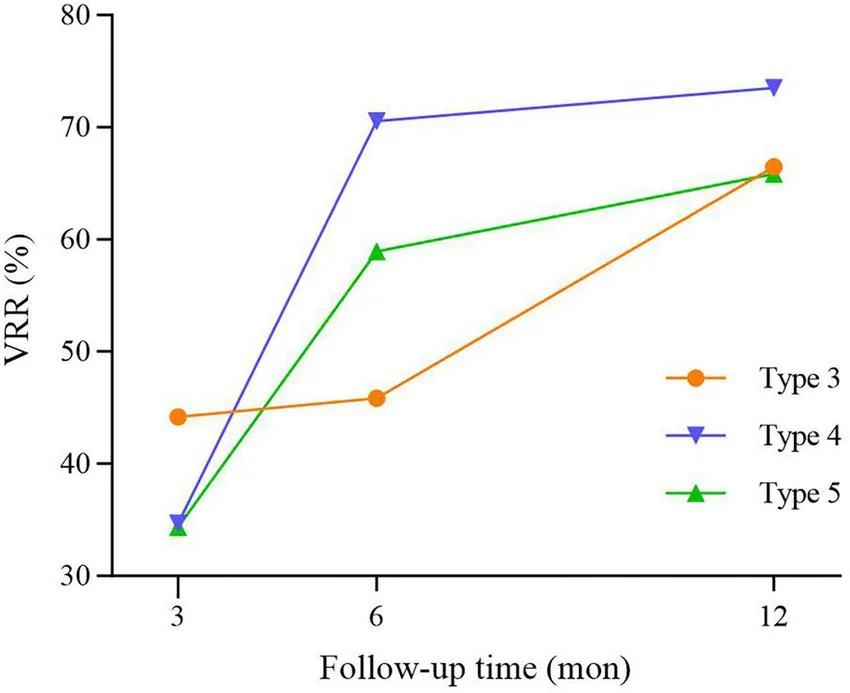
Volume reduction rate (VRR) by FIGO type in the MWA group. VRR, volume reduction rate.

Comparative analysis of volume changes across different FIGO classifications revealed that FIGO type 3 fibroids exhibited the most pronounced volume reduction at the 3-month follow-up, whereas type 4 fibroids demonstrated the greatest reduction at the 6- and 12-month follow-ups (Figure 5).

### 3.3 Comparison of perioperative outcomes between the two groups

Although no statistically significant difference was observed in median operative time between the MWA and LM groups (82.00 min vs. 90.00 min; *P* = 0.17), the MWA group demonstrated favorable outcomes in terms of shorter median postoperative hospital stay (2.00 days vs. 6.00 days; *P* < 0.001) and lower median hospitalization costs (12.92 k vs. 16.21 k; *P* = 0.01). Detailed results are summarized in Table 3.

**TABLE 3:** Perioperative outcomes between the two groups.

Perioperative outcomes	MWA group	LM group	*P*-value
Patient			
Operation time (min)^a^	82.00 (57.00, 96.00)	90.00 (75.00, 110.00)	0.17
Postoperative hospital stay (days)^a^	2.00 (1.00, 2.00)	6.00 (5.00, 6.50)	<0.001
Inpatient costs (k)^a^	12.92 (11.41, 16.19)	16.21 (14.34, 17.20)	0.01
Complication			
Major complication	0	0	/
Minor complication	4 (17.39)	5 (13.51)	0.72

^a^Data are medians with IQRs in parentheses. MWA, microwave ablation; LM, laparoscopic myomectomy.

### 3.4 Safety evaluation

No major complications, including adjacent organ injury, massive hemorrhage, or severe infection, were observed during or after the treatment in either group. The overall incidence of minor complications was comparable between groups (*P* = 0.72), as detailed in Table 3. In the MWA group, these included limb numbness (*n* = 1), vaginal discharge (*n* = 2), and peritoneal effusion (*n* = 1). In the LM group, complications comprised dysuria (*n* = 1), fever (*n* = 4), and chemosis (*n* = 1), with one patient experiencing both fever and chemosis. All complications resolved after symptomatic management.

## 4 Discussion

The principal therapeutic goal for uterine fibroids is to achieve symptomatic relief while preserving tissue integrity and minimizing iatrogenic injury(21, 22). In our cohort, MWA demonstrated comparable short-term efficacy and safety to LM, along with shorter postoperative hospital stay and lower inpatient costs. Additionally, the temporal pattern of fibroid volume reduction after MWA varied according to FIGO subtype.

While overall symptom resolution was comparable between groups, the LM group showed numerically better outcomes for bleeding-related symptoms and pelvic pressure. A possible explanation is that LM provides immediate relief by removing the fibroid and its mass effect, whereas MWA relies on gradual absorption or expulsion of necrotic tissue to achieve fibroid shrinkage (17). Consequently, the optimal therapeutic outcome from MWA typically requires a longer time to manifest, as has been suggested in previous studies (21, 23, 24). Xia et al.(23) reported that in 37 patients with uterine fibroids larger than 300 ml, the fibroid volume decreased from a preoperative value of 334.28 ml to 52.01 ml at 12 months, accompanied by a decline in symptom severity score from 32.70 to 12.64.

Consistent with the study by Jonsdottir et al. (25), which reported that the efficacy of MWA is correlated with the anatomical location of uterine fibroids, our analysis by FIGO subtype revealed distinct temporal patterns of volume reduction. Specifically, FIGO type 3 fibroids achieved the highest VRR at the 3-month assessment, while type 4 fibroids showed the best VRR at the 6- and 12-month follow-ups. One possible explanation for this difference is that type 3 fibroids, being in contact with the endometrium, may facilitate the natural expulsion of necrotic tissue through the uterine cavity, contributing to an earlier observed volume reduction. In contrast, the more favorable long-term outcomes observed in type 4 fibroids may be linked to their intramural location. Being enveloped by normal myometrium may not only facilitate a more thorough and safer initial ablation but also provide a vascularized environment that promotes post-ablation immune clearance and fibrotic repair, thereby contributing to sustained tissue shrinkage.

In our cohort, fibroid volume reduction was most rapid during the first 6 months, with a median VRR of 58.27%. This was lower than the 78.5% reported in a multicenter study of 311 patients and the 65.8% reported in another cohort with smaller baseline volumes (19, 21). One possible explanation is differences in fibroid location: Jonsdottir et al. (25) reported that at 6 months post-ablation, submucosal fibroids exhibited the highest VRR of 82%, followed by intramural fibroids at 66%, while subserosal fibroids showed the lowest reduction at 55%. Given that all fibroids in our cohort were intramural in location, the inclusion of submucosal fibroids in the other studies may partly explain their higher VRRs. Additionally, differences in baseline fibroid volume and ablation protocols across centers may also contribute. Nonetheless, the progressive volume reduction over time remains consistent across studies, supporting the durability of MWA efficacy.

Both MWA and LM demonstrated a favorable safety profile, with no serious complications occurring in either group. The overall incidence of minor adverse events was comparable, though their nature differed due to distinct mechanisms. Complications in the MWA group were primarily related to the local thermal effect and necrotic tissue expulsion, whereas those in the LM group stemmed from surgical manipulation and general anesthesia. Beyond safety, MWA was also associated with shorter postoperative hospitalization and lower inpatient costs in our cohort. This advantage is likely attributable to its technical features. FIGO type 3–5 fibroids are predominantly intramural and often enveloped by a pseudo-capsule, which acts as a thermal barrier that concentrates the ablative effect and protects adjacent tissue (22, 26). In addition, real-time ultrasound guidance allows precise targeting, avoiding surgical tissue trauma, while thermal coagulation minimizes intraoperative bleeding (4). Furthermore, MWA can be performed under intravenous anesthesia combined with local anesthesia at the puncture site (17), supporting its potential as an outpatient procedure in the future.

Therefore, for patients prioritizing minimal invasiveness and lower costs, MWA may represent a favorable alternative, whereas LM may remain the preferred option for those who prioritize complete fibroid resection. Moreover, fibroids are characterized by a high recurrence rate, with up to 20% of patients requiring reintervention within 11.4 years after initial myomectomy (27). In this context, MWA presents a favorable option, benefiting from its minimally invasive nature. Furthermore, as fibroid growth is hormonally driven and tends to regress after menopause (28, 29), MWA may be particularly suitable for premenopausal patients, helping control symptoms and facilitating a smooth transition into menopause.

To our knowledge, this is among the first studies to directly compare MWA and LM in a relatively homogeneous cohort of patients with FIGO type 3–5 fibroids, minimizing confounding by fibroid location and subtype and providing information on subtype-specific responses to MWA. Our study still has several limitations. First, as a retrospective investigation, the data collection and analysis may be influenced by potential selection bias and incomplete records. Second, the relatively small sample size and limited duration of follow-up may have compromised the statistical power, thereby restricting the capacity to adequately detect certain intergroup differences. Furthermore, impacts on fertility and pregnancy outcomes remain unclear and warrant future investigation. To address these limitations, prospective, multicenter randomized trials with extended follow-up are needed to provide higher-level evidence to guide clinical management.

## 5 Conclusion

In our study, both MWA and LM achieved comparable short-term symptom improvement and similar safety profiles for patients with FIGO type 3–5 uterine fibroids, with no major complications observed in either group. In addition, MWA was associated with significantly shorter hospital stay and lower treatment costs. Regarding fibroid volume reduction following MWA, the therapeutic response varied by subtype: FIGO type 3 fibroids exhibited more pronounced volume reduction at 3 months post-procedure, whereas type 4 fibroids showed a more sustained shrinkage trend at 6 and 12 months. Based on these findings, MWA may be preferred for patients seeking a minimally invasive approach with lower costs, while LM remains a reliable option for those prioritizing immediate and complete fibroid removal.

## Data Availability

The raw data supporting the conclusions of this article will be made available by the authors, without undue reservation.
